# LC-ESI-MS/MS Simultaneous Analysis Method Coupled with Cation-Exchange Solid-Phase Extraction for Determination of Pyrrolizidine Alkaloids on Five Kinds of Herbal Medicines

**DOI:** 10.1093/jaoacint/qsab098

**Published:** 2021-07-23

**Authors:** Se Hee Jeong, Eun Young Choi, Jonghwan Kim, Chulhyun Lee, Juhye Kang, Sooyeul Cho, Kyung Yuk Ko

**Affiliations:** National Institute of Food and Drug Safety Evaluation, Herbal Medicine Research Division, Osong-eup, Cheongju-si, Chungbuk 28159, Republic of Korea

## Abstract

**Background:**

Pyrrolizidine alkaloids (PAs) are naturally occurring plant toxins associated with potential hepatic and carcinogenic diseases in humans and animals. The concern over PAs has increased as the consumption of herbal medicines has increased.

**Objective:**

This study aimed to develop and validate a sensitive analytical method to determine 28 PAs in five herbal medicines using liquid chromatography (LC)-electrospray ionization (ESI)-tandem mass spectrometry (MS/MS). Additionally, this study identified and quantified the amount of PAs in 10 samples of each herbal medicine.

**Methods:**

The pretreatment in the proposed LC-MS/MS analysis comprised solvent extraction using 0.05M H_2_SO_4_ in 50% methanol and clean-up step using an mixed-mode cationic exchange (MCX)-solid-phase extraction (SPE) cartridge. The PA contents in herbal medicines were measured by using the developed method.

**Results:**

The proposed method had recoveries ranging from 72.5–123.7% for the Atractylodis Rhizoma Alba, 70.6–151.7% for Alba Chrysanthmi Flos, 80.6–130.9% for Leonuri Herba, 70.3–122.9% for Gastrodiae Rhizoma, and 67.1–106.9% for Glycyrrhizae Radix. Even though a few samples showed recoveries in unsatisfactory values, the proposed method indicated entirely sufficient recoveries and precision in most samples. In monitoring results, only Leonuri Herba contained two PAs, which indicated Retrorsine (4/10) of 84.7–120.9 μg/kg and Senkirkine (10/10) of 60.9–170.7 μg/kg.

**Conclusion:**

The results obtained from this study demonstrate that the proposed method is fit for purpose to determine 28 PAs in herbal medicines. Therefore it could serve as a regulatory method capable of being used for controlling the risks of PAs in certain medicinal plants and dietary supplements.

**Highlights:**

An LC-MS/MS method for the determination of 28 pyrrolizidine alkaloids in herbal medicines was developed and validated through this study. The proposed method is considered as an useful method for monitoring pyroolizidine alkaloids in herbal medicines.

Pyrrolizidine alkaloids (PAs) are secondary plant metabolites produced naturally for protection against herbivores in some plant families (1). PAs are known to be found in more than 12 plant families. Approximately 95% of PAs are found in plant families as follows: *Boraginaceae* (all genera), *Asteraceae* (subtribe *Senecioneae* and *Eupatorieae*), *Fabaceae* (subtribe *Crotalariaceae*, mainly genus *Crotalaria*) Orchidaceae (2–4). More than 660 different PAs and PA N-oxides have been identified in over 6000 plant species of these families (3). Their acute toxicity, genotoxicity, and carcinogenic potential in humans and animals have been known for decades (5–8). The International Agency for Research on Cancer (IARC) classifies Lasiocarpine, Monocrotaline, and Riddelline as “possibly carcinogenetic to humans (class 2B)” and Isatidine, Retrorsine, and Senkirkine, that have only limited evidence, as “not classifiable as to its carcinogenicity to humans (class 3)” (9, 10). The use of traditional medicines in developed countries is exponentially growing. The World Health Organization (WHO) estimates that the market for herbals and dietary supplements will increase to $5 trillion by 2050 (8, 11). As the raw plant materials are used to produce dietary supplements, and traditional medicines are widely distributed globally and may contain toxic PAs, the potential risks to human health of harmful side effects of PAs are a concern. There are several studies reporting on monitoring of PAs in Chinese medicinal plants (3, 12–15). For instance, Roeder (3) reported that 90 PAs were found in 38 traditional Chinese herbal medicines. However, they have not yet been well examined and characterized, even though many more Chinese herbal plants may contain PAs.

Due to the risk of overuse and high toxicity, the Herbal Medicinal Products Committee (HMPC) of the European Medicines Agency (EMA) has implemented a limit on intake of PAs from herbal medicinal products (i.e., 1 μg PAs per day) as a transitional measure for 3 years, after which the threshold will be set to 0.007 μg of 1,2-unsaturated PAs per kg body weight (i.e., 0.35 μg PAs per day for a 50 kg adult and 0.14 μg Pas per day for children) (16, 17). However, in 2019, the HMPC announced a consensus to extend the transitional period for 2 years (18, 19). BfArM (The Federal Institute for Drugs and Medical Devices, Germany) recommended the maximum daily dose of toxic PAs for internal use to be set at 1 μg at most 6 weeks per year and 0.1 μg without any limitations in the duration (20, 21). Furthermore, European Pharmacopoeia recently adopted a new chapter on PAs about general policies, including an analytical procedure for determining PAs and verification requirements (22). In Korea, the Ministry of Food and Drug Safety (MFDS) has applied the regulatory decision, since 2019, that the daily intake of PAs must not exceed 200 μg/kg in pollen products. However, a regulatory decision related to PA contamination in herbal medicines has not yet been established.

Various analytical methods have been developed for quantification of PAs present in herbal plants, dietary supplements, food products, and poisoned animals, as follows: Nuclear magnetic resonance (NMR) spectroscopy (23), enzyme-linked immunosorbent assay (ELISA) (13, 24), high-performance liquid chromatography (HPLC) (25), gas chromatography (GC)-mass spectrometry (MS) (26, 27), HPLC-mass spectrometry (MS) (28–31), and ultra-high performance liquid chromatography (UPLC)-quadrupole time-of-flight (Q-TOF) MS (32). As raw herbal medicines are used for herbal preparations, herbal medicinal products are comprised of multi-component mixtures which may interfere with the precise quantification of PAs. Verification of the analysis method is required for every herbal preparation or herbal drug, even if a validated method for determining PAs exists (33). Kopp et al. (34) explain that, for quantification of PAs in herbal medicine products, analytical techniques like GC-MS or liquid chromatography (LC)-tandem mass spectrometry (MS/MS) which have high sensitivity and selectivity are required to ensure PA levels are controlled in line with the limits concerning public health. They claim that LC-MS methods are the most suitable procedures for achieving precise quantification of PAs in herbal medicines. The EMA has recommended the LC-MS/MS (BfR-PA-Tea, 30) process developed by the BfR for determining PAs in herbal medicinal products (35).

To develop an LC-MS/MS analysis method, the optimal solvent, such as solid-phase extraction (SPE) cartridge, elution solution, and LC-MS/MS analysis conditions, should be established because the sample preparation and the analysis conditions of equipment can influence the recovery of the analytes. A validated LC-MS/MS analysis method is needed for the precise quantitative analysis of 28 PAs in herbal medicines, since the herbal medicines have various components with physicochemical characteristics as natural products. This study aimed to develop and validate a precise and convenient LC-MS/MS method for the determination of 28 PAs in five kinds of herbal medicines and investigate the contamination status of PAs using the proposed LC-MS/MS analysis method.

## Experimental

### Chemicals and Reagents

**Figure 1.**
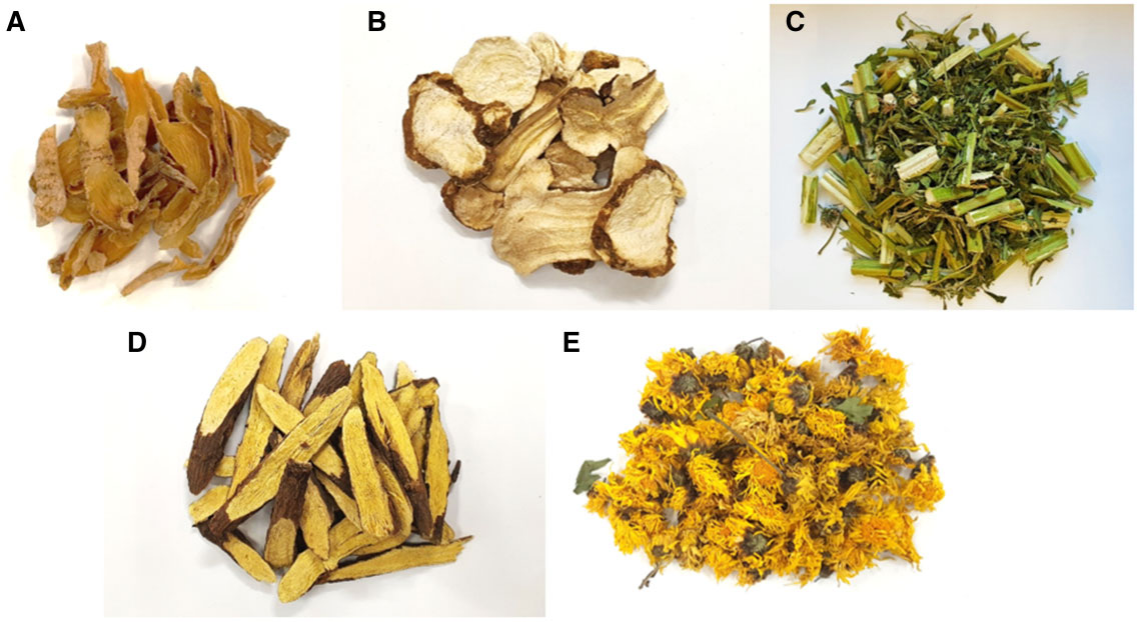
Pictures of five herbal medicine products used in this study. (A) Gastrodiae Rhizoma (*Gastrodia elata* Blume), (B) Atractylodis Rhizoma Alba (*Atractylodes japonica* Koidzumi), (C) Leonuri Herba (*Leonurus japonicus* Houttuyn), (D) Glycyrrhizae Radix et Rhizoma (*Glycyrrhiza uralensis* Fischer), (E) Chrysanthmi Flos (*Chrysanthemum morifolium* Ramatuelle).

The 28 PA standards used in the present study were purchased from Phytolab (Vestenbergsgreuth, Germany). Their abbreviations are shown in Table 1. Methanol (HPLC grade), ammonia solution (25%), and water (LC-MS grade) manufactured by Merck KGaA (Darmstadt, Germany), and ammonium formate, formic acid, and sulfuric acid (H_2_SO_4_, 98%), purchased from Sigma-Aldrich (St. Louis, MO, USA), were used. Through a review of literature related to PAs, various herbal medicines belonging to the plant family groups known to possess PAs were chosen. The five kinds of herbal medicines selected as target products in the first year of the MFDS project in Korea were Atractylodis Rhizoma Alba (*Atractylodes japonica* Koidzumi), Leonurus Herba *(Leonurus japonicus* Houttuyn), Gastrodiae Rhizoma (*Gastrodia elata* Blume), Glycyrrhizae Radix (*Glycyrrhiza uralensis* Fischer), and Chrysanthmi Flos (*Chrysanthemum morifolium* Ramatuelle) (Figure 1). The herbal medicines were purchased from a herbal medicinal store located in Daejeon, Korea. All samples were commercial herbal medicinal products manufactured in Korea. The majority of the herbal medicine samples were produced and imported from China, whereas a few samples were produced in Korea. Before carrying out any further experiments, the identities of the herbal medicine samples were confirmed through sensory tests performed by a specialist. Voucher specimens (PA-01–43) were deposited at the National Institute of Food and Drug Safety Evaluation (Cheongju, Republic of Korea).

**Table 1.** The chemical information of 28 PA compounds used in this study and the MRM conditions established for the LC-ESI-MS/MS analysis

No.	PA compounds	PAs abbr.	Retention time, min	Formula	CAS No.	Precursor ion [M + H]^+^	MRM ion transitions, *m/z*		Collision energy, eV	Transition ratio, %^a^
							Quantitative ion	Confirmation ion		
1	Echimidine	Em	19.56	C_20_H_31_NO_7_	520-68-3	398	120	220	–25	19
2	Echimidine N-oxide	EmN	19.33	C_20_H_31_NO_8_	41093-89-4	414	254	352	–17	27
3	Erucifoline	Er	6.35	C_18_H_23_NO_6_	40158-95-0	350	120	138	–31	52
4	Erucifoline N-oxide	ErN	7.40	C_18_H_23_NO_7_	123864-94-8	366	94	119	–25	70
5	Europine	Eu	7.25	C_16_H_27_NO_6_	570-19-4	330	138	156	–29	26
6	Europine N-oxide	EuN	8.18	C_16_H_27_NO_7_	65582-53-8	346	172	111	–28	20
7	Heliotrine	Hn	11.33	C_16_H_27_NOs	303-33-3	314	138	156	–44	20
8	Heliotrine N-oxide	HnN	13.10	C_16_H_27_NO_6_	6209-65-0	330	172	111	–31	25
9	Intermedine	Im	7.28	C_15_H_25_NO_5_	10286-06-0	300	94	138	–34	69
10	Intermedine N-oxide	ImN	8.87	C_15_H_25_NO_6_	95462-14-9	316	172	94	–25	53
11	Jacobine	Jb	7.31	C_18_H_25_NO_6_	6870-67-3	352	120	155	–31	76
12	Jacobine N-oxide	JbN	8.10	C_18_H_25_NO_7_	38710-25-7	368	296	120	–44	83
13	Lasiocarpine	Lc	20.23	C_21_H_33_NO_7_	303-34-4	412	120	336	–20	38
14	Lasiocarpine N-oxide	LcN	20.33	C_21_H_33_NO_8_	127-30-0	428	254	94	–28	46
15	Lycopsamine	La	7.67	C_15_H_25_NO_5_	10285-07-1	300	94	138	–27	61
16	Lycopsamine N-oxide	LaN	9.41	C_15_H_25_NO_6_	95462-15-0	316	172	94	–43	49
17	Monocrotaline	Mc	5.48	C_16_H_23_NO_6_	315-22-0	326	120	94	–27	46
18	Monocrotaline N-oxide	McN	7.06	C_16_H_23_NO_7_	35337-98-5	342	137	119	–20	44
19	Retrorsine	Re	10.38	C_18_H_25_NO_6_	480-54-6	352	120	138	–28	86
20	Retrorsine N-oxide	ReN	10.98	C_18_H_25_NO_7_	15503-86-3	368	94	118	–41	73
21	Senecionine	Sc	14.89	C_18_H_25_NO_5_	130-01-8	336	120	94	–31	68
22	Senecionine N-oxide	ScN	16.09	C_18_H_25_NO_6_	13268-67-2	352	94	118	–29	73
23	Seneciphylline	Sp	11.45	C_18_H_23_NO_5_	480-81-9	334	120	94	–26	76
24	Seneciphylline N-oxide	SpN	6.35	C_18_H_23_NO_6_	38710-26-8	350	120	94	–38	54
25	Senecivernine	Sv	14.22	C_18_H_25_NO_5_	72755-25-0	336	120	308	–28	66
26	Senecivernine N-oxide	SvN	15.11	C_15_H_25_NO_6_	101687-28-9	352	118	94	–9	106
27	Senkirkine	Sk	20.04	C_19_H_27_NO_6_	2318-18-5	366	168	122	–29	41
28	Trichodesmine	Td	10.41	C_18_H_27_NO_6_	548-90-3	354	222	120	–48	74

a The transition ratios were calculated by dividing the area of the confirmation ion by the area of the quantitation ion in the calibration samples.

### Standard Solution Preparation

The stock solutions of each PA standard were prepared to a concentration of 100 μg/mL in MeOH and stored at –20°C. The mixed PA standard solution was prepared by taking a part of the volume of each solution and mixing them with the solvent of 5% methanol. As this study conducted a matrix-matched analysis, the mixed PA standard solution was serially diluted with the blank sample solution, which did not contain any analytes. Through the preliminary test, the suitable concentrations in each PA were determined and the standard solution was prepared to be within the calibration concentration range as shown in Tables 2–6. The calibration curve was measured at five concentrations from the lowest concentration to its 40 times concentration. The working standard solutions were prepared separately for all different matrixes as the calibration ranges were different for the same PAs in different herbal matrixes.

**Table 2.** Linearities, LODs, LOQs, recoveries, intra-day and inter-day RSDs, and MEs obtained using the LC-ESI-MS/MS analysis method in Atractylodis Rhizoma Alba

No.	PAs	Cali. concn range, ng/mL	Linearity (r^2^)	LOD, μg/kg	LOQ, μg/kg	Recovery, % mean ±SD			Intra-day RSD, %	Inter-day RSD, % (*n* = 9)	Matrix effect
						Low level	Medium level	High level			
1	Echimidine	0.2–8	0.999	0.03	0.10	95.5 ± 2.7	93.7 ± 3.6	93.8 ± 4.5	3.8	6.1	88(–)^a^
2	Echimidine N-oxide	0.5–20	0.998	1.27	3.80	87.0 ± 1.7	86.4 ± 2.4	86.6 ± 1.0	1.9	6.5	86(–)
3	Erucifoline	1–40	0.999	0.37	1.10	99.6 ± 1.4	95.1 ± 3.7	94.8 ± 0.5	2.0	4.6	71(–)
4	Erucifoline N-oxide	1–40	0.999	0.70	2.10	102.7 ± 4.8	93.5 ± 6.0	93.3 ± 8.3	6.6	8.1	99(–)
5	Europine	1–40	0.995	0.10	0.30	83.4 ± 4.9	93.5 ± 16.2	97.4 ± 9.2	10.9	12.8	57(–)
6	Europine N-oxide	0.5–20	0.999	0.03	0.10	114.0 ± 8.0	111.5 ± 5.4	111.9 ± 5.1	5.4	5.8	57(–)
7	Heliotrine	0.2–8	0.999	0.03	0.10	103.4 ± 5.6	96.3 ± 7.8	96.7 ± 11.6	8.5	6.1	107(+)^b^
8	Heliotrine N-oxide	0.2–8	0.999	0.03	0.10	103.6 ± 3.5	96.5 ± 4.8	96.2 ± 5.7	4.8	5.3	97(–)
9	Intermedine	1–40	0.999	0.03	0.10	90.1 ± 4.7	92.6 ± 4.1	95.5 ± 4.2	4.7	8.5	70(–)
10	Intermedine N-oxide	0.5–20	0.999	0.07	0.20	93.9 ± 1.8	93.4 ± 5.0	93.5 ± 2.0	3.1	5.1	94(–)
11	Jacobine	1–40	0.999	2.17	6.50	96.1 ± 5.5	95.0 ± 3.7	95.6 ± 4.2	4.7	7.2	57(–)
12	Jacobine N-oxide	1–40	0.999	0.03	0.10	92.9 ± 5.8	95.0 ± 5.3	95.3 ± 4.5	5.5	3.3	82(–)
13	Lasiocarpine	0.5–20	0.999	0.03	0.10	93.2 ± 20.1	101.9 ± 16.9	107.8 ± 10.3	15.9	10.0	95(–)
14	Lasiocarpine N-oxide	2–80	0.999	0.53	1.60	123.7 ± 0.4	97.5 ± 24.0	96.2 ± 22.8	16.2	13.5	54(–)
15	Lycopsamine	0.5–20	0.999	0.03	0.10	118.3 ± 9.5	97.9 ± 16.2	97.9 ± 23.2	16.1	9.4	114(+)
16	Lycopsamine N-oxide	1–40	0.999	0.13	0.40	102.0 ± 9.2	93.4 ± 14.4	93.5 ± 16.3	14.0	7.5	92(–)
17	Monocrotaline	0.5–20	0.999	0.17	0.50	98.3 ± 7.6	96.2 ± 7.5	97.6 ± 10.2	8.6	8.2	46(–)
18	Monocrotaline N-oxide	1–40	0.999	0.13	0.40	99.5 ± 7.1	91.9 ± 8.6	93.0 ± 12.1	9.8	6.9	105(+)
19	Retrorsine	1–40	0.999	1.00	3.00	91.6 ± 4.3	92.0 ± 5.3	95.4 ± 7.7	6.2	8.6	99(–)
20	Retrorsine N-oxide	1–40	0.999	0.27	0.80	93.8 ± 4.2	94.4 ± 4.0	95.0 ± 3.4	4.1	6.2	107(+)
21	Senecionine	0.5–20	0.999	0.67	2.00	102.7 ± 4.4	93.4 ± 6.0	93.9 ± 8.1	6.4	6.2	109(+)
22	Senecionine N-oxide	1–40	0.999	0.43	1.30	98.8 ± 5.6	95.7 ± 6.5	97.0 ± 8.5	7.0	7.9	97(–)
23	Seneciphylline	1–40	0.999	0.83	2.50	89.3 ± 2.0	85.2 ± 2.0	84.0 ± 1.6	2.1	5.4	102(+)
24	Seneciphylline N-oxide	1–40	0.999	0.53	1.60	109.1 ± 5.9	98.1 ± 6.5	97.8 ± 9.5	7.2	5.4	78(–)
25	Senecivernine	0.5–20	0.999	0.50	1.50	98.6 ± 2.1	91.0 ± 2.2	89.6 ± 1.4	2.1	4.4	100(+)
26	Senecivernine N-oxide	0.5–20	0.999	0.10	0.30	77.1 ± 1.1	73.6 ± 2.5	72.5 ± 1.8	2.4	9.2	91(–)
27	Senkirkine	1–40	0.999	0.03	0.10	119.2 ± 3.4	102.5 ± 9.2	99.2 ± 13.3	8.4	3.9	56(–)
28	Trichodesmine	0.5–20	0.999	0.10	0.30	90.1 ± 2.9	92.9 ± 4.4	94.2 ± 4.8	4.3	3.8	91(–)

a – = Ion suppression.

b += Ion enhancement.

**Table 3.** Linearities, LOD, LOQ, recoveries, intra-day and inter-day RSDs, and matrix effect obtained by LC-ESI-MS/MS analysis method in Chrysanthmi Flos

No.	PAs	Cali. concn range, ng/mL	Linearity, r^2^	LOD, μg/kg	LOQ, μg/kg	Recovery, % (mean ±SD)			Intra-day RSD, %	Inter-day RSD, % (*n *= 9)	Matrix effect
						Low level	Medium level	High level			
1	Echimidine	0.5–20	0.999	0.23	0.70	81.0 ± 1.9	90.2 ± 4.7	100.7 ± 4.5	4.0	3.6	26(–)^a^
2	Echimidine N-oxide	1–40	0.999	0.87	2.60	77.7 ± 1.6	82.7 ± 1.3	84.8 ± 0.8	1.5	1.0	61(–)
3	Erucifoline	1–40	0.992	0.70	2.10	100.8 ± 5.7	95.8 ± 7.0	127.2 ± 7.9	6.7	6.2	50(–)
4	Erucifoline N-oxide	1–40	0.999	0.93	2.80	90.0 ± 1.9	84.2 ± 1.1	93.1 ± 0.8	1.4	2.0	78(–)
5	Europine	0.5–20	0.998	0.10	0.30	155.4 ± 6.0	102.3 ± 11.8	110.5 ± 3.4	6.2	7.4	181(+)^b^
6	Europine N-oxide	0.5–20	0.999	0.10	0.30	81.2 ± 3.8	83.6 ± 5.6	94.9 ± 1.5	4.3	2.6	93(–)
7	Heliotrine	0.1–4	0.999	0.03	0.10	94.6 ± 6.5	86.3 ± 3.2	97.1 ± 2.7	4.4	2.6	81(–)
8	Heliotrine N-oxide	0.5–20	0.999	0.07	0.20	89.2 ± 1.5	86.6 ± 0.9	93.2 ± 2.2	1.7	1.5	91(–)
9	Intermedine	1–40	0.999	0.03	0.10	84.9 ± 4.8	86.7 ± 5.3	103.5 ± 3.0	4.9	2.7	73(–)
10	Intermedine N-oxide	0.5–20	0.999	0.17	0.50	83.1 ± 3.1	87.5 ± 1.0	96.5 ± 1.1	2.0	1.7	91(–)
11	Jacobine	1–40	0.988	0.70	2.10	113.3 ± 6.0	88.0 ± 8.0	126.7 ± 11.0	7.7	5.9	57(–)
12	Jacobine N-oxide	0.5–20	0.999	0.13	0.40	118.9 ± 17.3	100.4 ± 2.8	103.6 ± 1.4	6.2	5.3	92(–)
13	Lasiocarpine	0.5–20	0.999	0.50	1.50	121.4 ± 8.0	75.8 ± 5.9	69.7 ± 7.3	8.3	7.5	13(–)
14	Lasiocarpine N-oxide	1–40	0.999	1.43	4.30	91.1 ± 12.7	68.7 ± 8.8	77.3 ± 11.5	13.9	10.6	17(–)
15	Lycopsamine	0.5–20	0.999	0.03	0.10	98.2 ± 3.8	90.3 ± 1.8	107.1 ± 2.2	2.6	1.8	67(–)
16	Lycopsamine N-oxide	2–80	0.992	0.27	0.80	76.8 ± 7.3	73.7 ± 6.1	89.4 ± 3.2	7.1	3.7	60(–)
17	Monocrotaline	1–40	0.999	0.03	0.10	102.4 ± 1.0	89.4 ± 2.9	101.8 ± 2.9	2.3	2.2	29(–)
18	Monocrotaline N-oxide	1–40	0.999	0.67	2.00	87.7 ± 4.5	84.8 ± 2.5	91.8 ± 2.2	3.5	2.4	67(–)
19	Retrorsine	1–40	0.986	1.00	3.00	106.2 ± 4.0	101.7 ± 8.4	146.2 ± 8.4	5.9	4.6	70(–)
20	Retrorsine N-oxide	1–40	0.999	0.87	2.60	82.5 ± 4.0	87.3 ± 2.1	95.9 ± 1.2	2.8	2.1	92(–)
21	Senecionine	0.5–20	0.983	1.40	4.20	120.6 ± 4.5	103.5 ± 8.6	151.7 ± 10.0	6.2	5.1	64(–)
22	Senecionine N-oxide	1–40	0.999	1.77	5.30	88.2 ± 4.4	83.0 ± 3.9	94.3 ± 4.3	4.8	3.3	65(–)
23	Seneciphylline	2–80	0.991	3.37	10.10	117.8 ± 7.4	100.0 ± 7.3	138.3 ± 8.4	6.6	4.2	53(–)
24	Seneciphylline N-oxide	1–40	0.992	0.80	2.40	99.4 ± 3.2	94.6 ± 9.6	130.2 ± 6.8	6.2	5.1	48(–)
25	Senecivernine	0.5–20	0.989	1.23	3.70	115.3 ± 6.4	101.2 ± 6.2	137.7 ± 7.1	5.6	5.7	60(–)
26	Senecivernine N-oxide	1–40	0.999	0.23	0.70	77.2 ± 0.8	85.6 ± 3.4	93.0 ± 1.8	2.1	1.6	92(–)
27	Senkirkine	0.5–20	0.999	0.47	1.40	74.0 ± 2.7	70.6 ± 10.4	76.6 ± 16.3	13.2	12.2	8(–)
28	Trichodesmine	0.5–20	0.999	0.43	1.30	90.3 ± 1.3	89.6 ± 4.0	100.0 ± 1.0	2.3	1.7	66(–)

a – = Ion suppression.

b + = Ion enhancement.

**Table 4.** Linearities, LOD, LOQ, recoveries, intra-day and inter-day RSDs, and MEs obtained by the LC-ESI-MS/MS analysis method in Leonurus Herba

No.	PAs	Cali. concn range, ng/mL	Linearity, r^2^	LOD, μg/kg	LOQ, μg/kg	Recovery, % (mean ±SD)			Intra-day RSD, %	Inter-day RSD, % (*n *= 9)	Matrix effect
						Low level	Medium level	High level			
1	Echimidine	1–40	0.998	0.03	0.10	123.9 ± 16.2	93.3 ± 11.2	104.2 ± 10.3	11.7	19.3	28(–)^a^
2	Echimidine N-oxide	0.5–20	0.999	0.30	0.90	112.3 ± 0.3	96.4 ± 3.4	94.7 ± 3.9	2.6	4.9	87(–)
3	Erucifoline	1–40	0.999	0.10	0.30	115.3 ± 0.9	107.8 ± 3.3	88.1 ± 4.1	2.8	17.2	113(+)^b^
4	Erucifoline N-oxide	1–40	0.999	0.40	1.20	101.9 ± 3.2	95.3 ± 2.1	99.2 ± 4.3	3.2	2.6	97(–)
5	Europine	1–40	0.999	0.03	0.10	128.4 ± 3.5	99.2 ± 4.8	95.2 ± 2.4	3.4	7.8	451(+)
6	Europine N-oxide	1–40	0.999	0.07	0.20	116.6 ± 1.9	102.6 ± 1.3	99.2 ± 2.1	1.7	1.8	82(–)
7	Heliotrine	1–40	0.999	0.03	0.10	118.8 ± 6.9	93.7 ± 5.1	93.9 ± 1.7	4.4	6.7	66(–)
8	Heliotrine N-oxide	1–40	0.999	0.03	0.10	121.2 ± 6.6	99.2 ± 4.9	97.7 ± 1.4	3.9	4.2	93(–)
9	Intermedine	1–40	0.999	0.03	0.10	119.4 ± 5.9	105.6 ± 3.2	90.0 ± 3.4	3.9	8.0	73(–)
10	Intermedine N-oxide	0.5–20	0.999	0.07	0.20	124.6 ± 8.6	103.4 ± 4.7	96.5 ± 2.5	4.6	4.6	121(+)
11	Jacobine	1–40	0.999	1.23	3.70	113.0 ± 1.0	106.5 ± 1.9	88.1 ± 4.1	2.5	16.0	80(–)
12	Jacobine N-oxide	1–40	0.999	0.07	0.20	108.2 ± 2.8	101.2 ± 0.7	93.6 ± 1.6	1.7	4.9	102(+)
13	Lasiocarpine	1–40	0.998	0.10	0.30	116.4 ± 33.4	95.6 ± 19.9	91.2 ± 18.4	23.2	13.0	11(–)
14	Lasiocarpine N-oxide	1–40	0.997	0.70	2.10	98.6 ± 19.4	88.9 ± 17.7	95.9 ± 18.7	19.7	9.4	10(–)
15	Lycopsamine	1–40	0.998	0.03	0.10	130.9 ± 4.0	100.8 ± 3.5	91.2 ± 1.4	2.7	10.6	71(–)
16	Lycopsamine N-oxide	1–40	0.999	0.17	0.50	87.8 ± 10.3	84.7 ± 8.3	105.2 ± 12.4	11.1	11.6	40(–)
17	Monocrotaline	0.5–20	0.999	0.13	0.40	111.8 ± 2.5	106.0 ± 1.9	100.8 ± 3.4	2.4	11.4	46(–)
18	Monocrotaline N-oxide	0.5–20	0.999	0.30	0.90	101.5 ± 3.5	94.1 ± 3.8	101.6 ± 4.1	3.8	6.6	70(–)
19	Retrorsine	0.5–20	0.999	0.83	2.50	123.7 ± 6.9	112.7 ± 2.9	94.7 ± 5.1	4.5	19.1	90(–)
20	Retrorsine N-oxide	0.5–20	0.999	0.27	0.80	104.8 ± 2.8	98.9 ± 0.7	96.5 ± 5.4	3.0	7.9	90(–)
21	Senecionine	0.5–20	0.999	1.07	3.20	109.9 ± 4.8	98.3 ± 3.5	81.6 ± 3.6	4.1	21.7	77(–)
22	Senecionine N-oxide	1–40	0.999	0.77	2.30	108.0 ± 9.2	95.3 ± 6.8	103.0 ± 2.9	6.2	9.2	57(–)
23	Seneciphylline	0.5–20	0.999	1.83	5.50	105.3 ± 8.7	98.7 ± 1.2	83.1 ± 6.7	5.9	25.7	66(–)
24	Seneciphylline N-oxide	1–40	0.999	0.20	0.60	117.1 ± 1.7	107.9 ± 1.8	92.0 ± 2.7	2.0	19.5	87(–)
25	Senecivernine	1–40	0.999	1.40	4.20	107.1 ± 5.3	97.2 ± 1.2	80.6 ± 2.6	3.2	20.3	71(–)
26	Senecivernine N-oxide	0.5–20	0.999	0.30	0.90	109.4 ± 2.4	102.5 ± 3.0	96.7 ± 1.2	2.1	4.8	119(+)
27	Senkirkine	0.1–5	0.998	0.03	0.10	103.6 ± 9.2	123.4 ± 11.1	108.0 ± 19.5	12.0	19.7	8(–)
28	Trichodesmine	0.5–20	0.999	0.10	0.30	95.0 ± 4.9	94.1 ± 1.8	93.6 ± 2.3	3.2	8.0	80(–)

a – = Ion suppression.

b + = Ion enhancement.

**Table 5.** Linearities, LOD, LOQ, recoveries, intra-day and inter-day RSDs, and MEs obtained by the LC-ESI-MS/MS analysis method in Gastrodiae Rhizoma

No.	PAs	Cali. concn range, ng/mL	Linearity, r^2^	LOD, μg/kg	LOQ, μg/kg	Recovery, % (mean ±SD)			Intra-day RSD, %	Inter-day RSD, % (*n *= 9)	Matrix effect
						Low level	Medium level	High level			
1	Echimidine	0.5–20	0.999	0.03	0.10	119.1 ± 8.9	95.8 ± 10.7	85.3 ± 2.5	7.2	11.2	53(–)^*^
2	Echimidine N-oxide	1–40	0.998	1.00	3.00	101.7 ± 2.3	96.4 ± 1.5	91.9 ± 1.0	1.6	3.3	83(–)
3	Erucifoline	1–40	0.999	0.60	1.80	88.2 ± 0.8	95.7 ± 1.6	98.1 ± 0.7	1.1	7.4	84(–)
4	Erucifoline N-oxide	1–40	0.999	0.77	2.30	84.5 ± 2.3	87.9 ± 2.4	89.4 ± 1.4	2.3	2.1	113(+)^**^
5	Europine	0.5–20	0.999	0.20	0.60	81.3 ± 3.1	87.2 ± 3.1	89.8 ± 1.3	2.9	5.2	48(–)
6	Europine N-oxide	0.2–8	0.999	0.03	0.10	96.5 ± 4.6	101.4 ± 4.7	100.3 ± 1.7	3.7	6.7	55(–)
7	Heliotrine	0.1–4	0.999	0.13	0.40	92.1 ± 7.3	80.4 ± 6.9	85.1 ± 1.0	5.9	13.3	73(–)
8	Heliotrine N-oxide	0.5–20	0.999	0.10	0.30	92.2 ± 6.1	86.5 ± 5.0	91.0 ± 2.2	4.9	5.4	105(+)
9	Intermedine	1–40	0.999	0.10	0.30	90.0 ± 1.6	95.3 ± 2.1	95.6 ± 0.8	1.6	7.0	67(–)
10	Intermedine N-oxide	0.5–20	0.999	0.10	0.30	93.9 ± 2.4	95.1 ± 2.5	94.2 ± 1.1	2.1	6.6	111(+)
11	Jacobine	1–40	0.999	1.03	3.10	83.0 ± 3.9	93.5 ± 4.5	95.5 ± 1.2	3.6	7.8	60(–)
12	Jacobine N-oxide	0.5–20	0.999	0.03	0.10	84.1 ± 1.4	88.9 ± 1.7	89.3 ± 0.2	1.3	9.0	89(–)
13	Lasiocarpine	0.5–20	0.998	0.17	0.50	75.1 ± 10.7	77.6 ± 9.7	82.5 ± 5.5	11.1	19.0	7(–)
14	Lasiocarpine N-oxide	1–40	0.999	1.40	4.20	102.3 ± 14.5	93.4 ± 8.0	92.5 ± 6.1	9.8	6.5	3(–)
15	Lycopsamine	0.5–20	0.999	0.10	0.30	94.0 ± 8.2	86.0 ± 7.3	89.9 ± 3.9	7.2	6.6	111(+)
16	Lycopsamine N-oxide	2–80	0.999	0.20	0.60	79.2 ± 8.7	88.4 ± 6.9	94.7 ± 4.7	7.9	10.5	87(–)
17	Monocrotaline	0.1–4	0.999	0.30	0.90	84.5 ± 6.8	93.5 ± 3.1	97.6 ± 2.2	4.6	8.3	71(–)
18	Monocrotaline N-oxide	1–40	0.999	0.43	1.30	70.3 ± 4.6	83.0 ± 4.1	89.6 ± 1.9	4.5	2.8	112(+)
19	Retrorsine	1–40	0.999	0.27	0.80	81.7 ± 1.7	90.3 ± 2.4	95.0 ± 0.6	1.8	3.6	78(–)
20	Retrorsine N-oxide	1–40	0.999	0.27	0.80	88.5 ± 2.8	90.0 ± 2.8	92.1 ± 0.7	2.4	1.7	119(+)
21	Senecionine	0.5–20	0.999	3.03	9.10	95.8 ± 5.6	93.7 ± 2.9	94.9 ± 1.9	3.6	8.7	81(–)
22	Senecionine N-oxide	1–40	0.998	0.20	0.60	103.6 ± 10.7	86.9 ± 10.4	89.6 ± 4.3	8.9	10.6	87(–)
23	Seneciphylline	2–80	0.999	0.13	0.40	79.9 ± 2.8	80.8 ± 3.1	85.7 ± 0.2	2.5	4.9	104(+)
24	Seneciphylline N-oxide	1–40	0.999	0.60	1.80	83.7 ± 2.9	90.3 ± 2.4	93.6 ± 0.8	2.4	1.5	84(–)
25	Senecivernine	0.5–20	0.999	0.37	1.10	90.8 ± 3.4	91.2 ± 2.6	94.3 ± 2.4	3.0	6.4	108(+)
26	Senecivernine N-oxide	1–40	0.999	0.13	0.40	85.4 ± 1.9	93.7 ± 2.3	92.2 ± 1.8	2.2	5.2	103(+)
27	Senkirkine	5–200	0.999	0.23	0.70	122.9 ± 11.8	85.6 ± 12.6	81.1 ± 2.1	9.0	15.3	1(–)
28	Trichodesmine	0.5–20	0.999	0.07	0.20	81.8 ± 3.5	92.5 ± 2.6	97.7 ± 1.3	2.8	2.0	83(–)

a – = Ion suppression.

b + = Ion enhancement.

**Table 6.** Linearities, LOD, LOQ, recoveries, intra-day and inter-day RSDs, and MEs obtained by the LC-ESI-MS/MS analysis method in Glycyrrhizae Radix et Rhizoma

No.	PAs	Cali. concn range, ng/mL	Linearity, r^2^	LOD, μg/kg	LOQ, μg/kg	Recovery, % (mean ±SD)			Intra-day RSD, %	Inter-day RSD, % (*n *= 9)	Matrix effect
						Low level	Medium level	High level			
1	Echimidine	0.5–20	0.999	0.03	0.10	84.6 ± 2.7	89.7 ± 0.5	92.5 ± 1.3	1.7	5.5	67(–)^a^
2	Echimidine N-oxide	0.5–20	0.999	1.53	4.60	93.5 ± 4.2	85.7 ± 2.3	84.1 ± 1.5	3.0	6.9	85(–)
3	Erucifoline	1–40	0.999	0.33	1.00	78.3 ± 1.4	84.1 ± 1.6	88.4 ± 2.7	2.3	3.9	70(–)
4	Erucifoline N-oxide	0.5–20	0.999	0.33	1.00	103.2 ± 1.0	96.5 ± 1.0	94.6 ± 1.0	0.9	4.1	88(–)
5	Europine	0.5–20	0.999	0.10	0.30	85.2 ± 16.1	92.7 ± 16.2	94.7 ± 16.8	18.0	9.2	51(–)
6	Europine N-oxide	1–40	0.999	0.03	0.10	93.4 ± 1.9	95.9 ± 0.8	92.5 ± 0.5	1.2	5.5	63(–)
7	Heliotrine	0.5–20	0.999	0.03	0.10	97.3 ± 1.3	92.4 ± 0.7	90.9 ± 0.9	1.1	4.2	102(+)^b^
8	Heliotrine N-oxide	0.5–20	0.999	0.03	0.10	104.2 ± 0.9	96.0 ± 1.2	93.0 ± 1.1	1.1	4.0	100(+)
9	Intermedine	1–40	0.999	0.03	0.10	87.2 ± 2.1	91.8 ± 0.3	93.9 ± 0.4	1.1	5.4	77(–)
10	Intermedine N-oxide	0.5–20	0.999	0.03	0.10	94.1 ± 2.9	94.8 ± 1.3	94.7 ± 2.4	2.3	3.8	93(–)
11	Jacobine	1–40	0.999	1.10	3.30	77.6 ± 2.4	85.7 ± 0.4	89.1 ± 0.8	1.5	8.7	62(–)
12	Jacobine N-oxide	0.5–20	0.999	0.13	0.40	96.7 ± 1.0	98.1 ± 2.0	95.6 ± 0.4	1.2	2.5	80(–)
13	Lasiocarpine	1–40	0.999	0.47	1.40	99.8 ± 2.4	73.8 ± 8.9	67.1 ± 0.2	4.9	9.1	7(–)
14	Lasiocarpine N-oxide	5–200	0.999	3.50	10.50	106.9 ± 23.2	83.9 ± 13.2	82.8 ± 13.9	18.1	17.1	7(–)
15	Lycopsamine	0.5–20	0.999	0.03	0.10	99.0 ± 1.2	93.0 ± 2.9	91.1 ± 2.5	2.4	3.7	86(–)
16	Lycopsamine N-oxide	0.5–20	0.999	0.10	0.30	99.5 ± 2.7	95.1 ± 1.8	93.6 ± 1.3	2.0	6.3	88(–)
17	Monocrotaline	0.5–20	0.999	0.13	0.40	95.5 ± 2.9	92.8 ± 2.4	93.6 ± 2.5	2.8	2.6	76(–)
18	Monocrotaline N-oxide	1–40	0.999	0.23	0.70	90.5 ± 1.0	92.4 ± 2.2	93.3 ± 1.5	1.7	4.6	96(–)
19	Retrorsine	0.5–20	0.999	0.17	0.50	90.9 ± 0.8	88.2 ± 0.5	88.4 ± 0.6	0.7	2.8	93(–)
20	Retrorsine N-oxide	1–40	0.999	0.13	0.40	85.5 ± 4.0	93.1 ± 3.0	94.4 ± 2.3	3.4	4.8	112(+)
21	Senecionine	0.5–20	0.999	1.60	4.80	81.5 ± 1.2	77.1 ± 0.4	76.6 ± 0.3	0.8	4.1	101(+)
22	Senecionine N-oxide	1–40	0.999	0.37	1.10	85.3 ± 2.3	90.4 ± 1.0	91.3 ± 1.7	1.9	5.5	77(–)
23	Seneciphylline	1–40	0.999	0.20	0.60	78.4 ± 3.7	78.1 ± 3.1	81.3 ± 3.7	4.4	3.4	99(–)
24	Seneciphylline N-oxide	1–40	0.999	0.50	1.50	95.2 ± 2.3	87.3 ± 0.8	88.1 ± 0.9	1.5	2.0	75(–)
25	Senecivernine	0.5–20	0.999	0.57	1.70	80.5 ± 1.8	77.6 ± 1.5	78.4 ± 1.9	2.4	4.0	94(–)
26	Senecivernine N-oxide	0.5–20	0.999	0.07	0.20	95.4 ± 2.3	93.3 ± 1.3	93.4 ± 1.7	1.9	4.2	91(–)
27	Senkirkine	5–200	0.999	0.10	0.30	87.9 ± 7.9	83.1 ± 3.5	71.8 ± 6.3	7.3	13.9	9(–)
28	Trichodesmine	0.5–20	0.999	0.10	0.30	95.2 ± 3.7	90.1 ± 4.5	91.0 ± 3.9	4.4	2.7	89(–)

a – = Ion suppression.

b + = Ion enhancement.

### Sample Preparation

Before grinding the herbal medicines, the samples (600 g) were divided into four equal parts to make them homogeneous. One of the parts of herbal medicine samples was ground into powder using a grinder (KSP-35, Koreamedi, Korea). The powder was weighed up to 2.0 g and mixed with 40 ml of extraction solution and 50% MeOH solution containing 50 mM sulfuric acid. After the addition of MeOH, the solutions were shaken for 30 min at room temperature to extract the PA compounds from the herbal medicines using an orbital shaker (SH30t, FINEPCR, Korea). After the extraction, the samples were centrifuged at 3320 × *g* for 10 min, and the supernatant was then collected. The investigators used MCX-SPE cartridges (Waters Oasis, Waters Corp., Milford, MA USA) to remove unnecessary materials contained in the supernatant. After the activation of the MCX-SPE cartridges, the solution preparation was completed by adding 3 mL of MeOH following 3 mL of distilled water. A part of the supernatant (2 mL) was loaded on the cartridge, and the cartridge was washed with 4 mL distilled water. Then, the solvent remaining in the cartridge was removed by pressure. After that, the elution solution (4 mL) mixed with the NH_4_OH and MeOH solutions (1:4, v/v) was added to elute the target PA compounds. The eluted solutions were evaporated at 50–55°C under nitrogen gas. After the nitrogen concentration, the residue was reconstituted with 1 mL of methanol-water (5:95, v/v). Last, the solution was filtered by a syringe filter (0.22 μm) and used as a sample solution for the LC-MS/MS analysis.

### LC-MS/MS Analysis

This study referred to the BfR test method (30), and the analysis procedure was developed by another laboratory in MFDS to create a more sensitive simultaneous analysis procedure for 28 PAs in herbal medicines. The LC-MS/MS system was coupled to a Shimadzu Nexera X2 LC-30AD, Shimadzu LCMS-8060 spectrometer in ESI positive ionization mode, and Shimadzu lab solution system (Shimadzu, Kyoto, Japan). The chromatographic separation was performed on a Shim-pack GIST-C18 (2.1 mm × 150 mm, 2 μm), and the column was maintained at 40°C. The mobile phases consisted of eluent A (0.1% formic acid in 5 mM ammonium formate) and eluent B (0.1% formic acid plus 5 mM ammonium formate in 100% methanol). A binary gradient profile was achieved as follows: 1.5 mins, 1% B; 1.5–3.0 mins, 1–15% B; 3.0–18.0 mins, 15–30% B; 18.0–19.0 mins, from 30 to 95% B with linear increase; 19.0–21.0 mins, held at 95% B; 21.1 mins, returned to 1% B. Re-equilibration between each run was 3.0 min. The injection volume was 5 μL, and the flow rate was maintained at 0.3 mL/min. The MS was performed in the positive-ion mode of the ESI source using Shimadzu lab solutions. Moreover, the mass spectrometer instrument and parameters were set as follows: drying gas temperature, 300°C; drying gas flow, 5.0 L/min; nebulizer pressure, 3 L/min; heat block temperature, 400°C; interface temperature, 400°C; and nebulizing gas flow, 15 L/min. Nitrogen was used as the drying and nebulizing gas. The multiple reaction monitoring (MRM) for all scan transitions was conducted on positive ion mode and at dwell time 13. The MRM conditions, including optimal precursor, ionic products, and collision energy in each PA, were newly obtained by the infusion of 10 ng/mL reference solutions of the targeted compounds to the LC-MS/MS system used in this study. The MRM mode determined in this study was confirmed to have high sensitivity for the 28 PAs in the LC-MS/MS analysis. The MRM values used in this study are displayed in Table 1.

### Matrix Effect

This study evaluated the matrix effect before the performance of the validation study. The matrix effect was quantitatively assessed by comparing the response of the analyte in the standard solution itself to that of the blank sample solution spiked with the analyte at the same concentration. The standard solution was prepared by dilution of the mixed stock PA standard solution with 5% MeOH, whereas the other standard solution was prepared by spiking the mixed stock standard solution to the blank sample solution. The blank sample solutions were prepared by the same pretreatment method described above. The calibration curves were achieved from five-point concentrations at the ranges shown in Tables 2–6. The matrix effect can be evaluated by the following formula (36): Matrix effect (ME, %) = (slope of the calibration curve in matrix/slope of the calibration curve insolvent) × 100.

### Method Validation

The analytical method was validated in terms of LOD, LOQ, linearity, reproducibility, repeatability (precision), and recovery (accuracy) as recommended by the AOAC guidelines (37). The validation study was conducted by a matrix-matched analysis against 28 PAs in herbal medicines and designed to be obtained using three different individual samples per matrix. The linearity was obtained by plotting the peak area of the research against the analyte concentration, which was assessed by the coefficients of determination (r^2^). Linearity was evaluated by the calibration curves obtained at the concentration range as shown in Tables 2–6 using the mixed 28 PA standard solutions with five calibration points. For the quantification, matrix-matched calibration curves in the concentration range were used. The LOD and LOQ were measured using the signal-to-noise ratio obtained after injection of the sample solution prepared by pretreating samples spiked with the standard solution with a concentration range.

For measuring the accuracy and precision against most PAs that were not contained in Farfarae Flos or Lithospermi Radix, the mixed PA standard with three different concentration levels (low, medium, and high) were spiked to the herbal medicine powers (2.0 g) to be 2, 5, and 10 times the lowest concentration at the range in each PA as shown in Tables 2–6, respectively. After conducting pretreatment with the spiked herbal medicine samples, the quantification analysis using LC-MS/MS was performed in triplicate with the sample solutions acquired from the pretreatment. For the quantification analysis, matrix-matched analysis was performed at the concentration range of each PA in herbal medicine samples. The intra-day precision (repeatability) and accuracy (recovery rates) were assessed through the analysis in triplicate on a single day. Contrastingly, the inter-day precision (reproducibility, RSD_r_) and accuracy were measured by the analysis in triplicate over three consecutive days (*n* = 9). Cross-validation of the proposed method was also carried out by the same procedure at the Technical Research Center, Shimadzu Scientific Korea.

### System Suitability

As an essential part of the LC method development, system suitability was ensured to interpret the chromatographic performance of the LC instrument. The resolution, tailing factor, the number of theoretical plates, peak width, and height equivalent to a theoretical plate were calculated via Shimadzu LC LabSolutions 5.86 SP1 data integration software. Also, the system suitability test was performed by injecting five replicates of the mixed standard solution with constant concentration and evaluated by the RSD of the peak area values.

### Monitoring of PAs in Herbal Medicines

This study attempted to measure the amount of 28 PAs in herbal medicinal samples. Ten samples of each herbal medicine, including Glycyrrhizae Radix et Rhizoma, Atractylodis Rhizoma Alba, Leonurus Herb, Gastrodiae Rhizoma, and Chrysanthmi Flos, were analyzed to determine the content of 28 PAs. Quantitative analysis was conducted as the matrix-matched analysis. In the quantitative analysis, the MRM conditions of each PA established in the present study for the LC-MS/MS simultaneous analysis were used. The contents of PAs in each sample were determined with triplicate samples (*n* = 3).

## Results and Discussion

### Optimization of the Analysis Method

**Figure 2.**
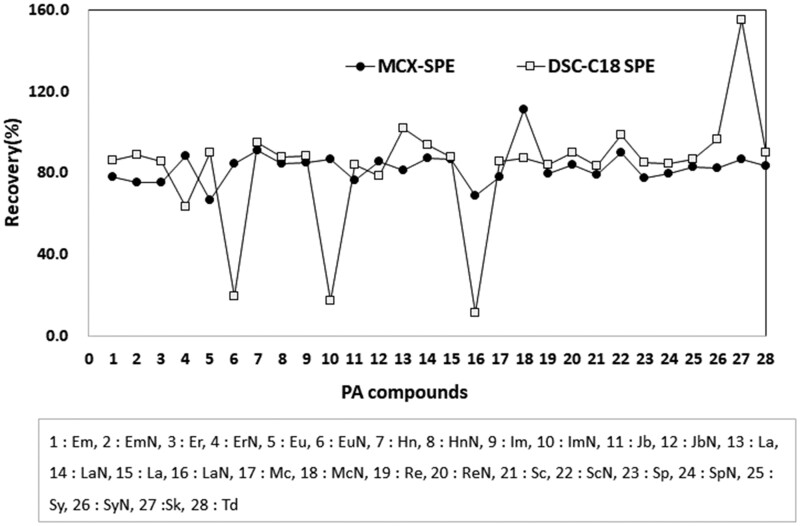
Comparison of the recovery (%) of the 28 PAs obtained by MCX-SPE and those by DSC-C18 SPE cartridges employed in the pretreatment processing to separate them from the herbal medicines.

This study was to develop an efficient analysis method for the determination of 28 PAs in herbal medicines by referring appropriately to the BfR (BfR-PA-Tea) and the MFDS method (MFDS-PA-Tea) developed by other laboratories in MFDS (unpublished data). Before developing the test method, this study tried to apply the BfR method, which was developed to determine 28 PAs in tea, for detection of PAs in herbal medicine samples. As a result, some PAs among the 28 PAs were revealed to have low recovery. Therefore, this study searched for more appropriate extraction solvent, cartridge for cleanup, elution solution, etc. to acquire appropriate recovery of 28 PAs in herbal medicine samples. The proposed LC-MS/MS used 0.05M H_2_SO_4_ in 50% MeOH as an extraction solution, instead of 0.05M H_2_SO_4_ (20 mL) employed in the BfR method. Additionally, the loading solution for purification was more easily prepared in the proposed method as it excluded the neutralization process from the BfR method. This study compared the recoveries of PAs obtained using a DSC-C18 employed in the BfR method to those obtained using the MCX-SPE cartridge in Chrysanthmi Flos. As shown in Figure 2, when the DSC-C18 SPE cartridge was used, even if the most PAs showed approximately 80% recoveries, three PAs (Europine N-oxide, Intermedine N-oxide, and Lycopsamine N-oxide) indicated very low recovery rates (<40%). Also, Senkirkin revealed a high recovery rate, displaying approximately 150%. However, the MCX-SPE cartridge showed an entirely satisfactory recovery in 28 PAs. Based on these results, the proposed analysis method selected the cation-exchange MCX-SPE cartridge to remove unnecessary components which could interfere with the precise quantitative analysis, instead of the DSC-C18 SPE cartridge.

The MFDS-PA-Tea method determining 21 PAs in food samples (unpublished data) uses 5.0% NH_4_OH solution in MeOH as elution solution for purification process using an MCX-SPE cartridge. This study used 2.5% NH_4_OH in MeOH for improvement of recovery because a part of PAs indicated a low recovery in using 5.0% NH_4_OH solution in MeOH. We assumed that the PAs trapped in the cartridge are better eluted, as the ammonia concentration increases (data not given). In general, it is considered to be important to establish LC-MS/MS analysis conditions for greater separation capacity in a developing LC-MS/MS method. This study referred to the LC-MS/MS conditions of the MFDS-PA-Tea method. In the initial step, we found that Echimidine N-oxide was not successfully separated from the peak of a component eluted at a similar time. When the eluent B solution was adjusted to that of the BfR method (0.1% formic acid and 5 mM ammonium formate in 100% methanol), the peak of Echimidine N-oxide was well separated from other peaks (data not given).

**Figure 3.**
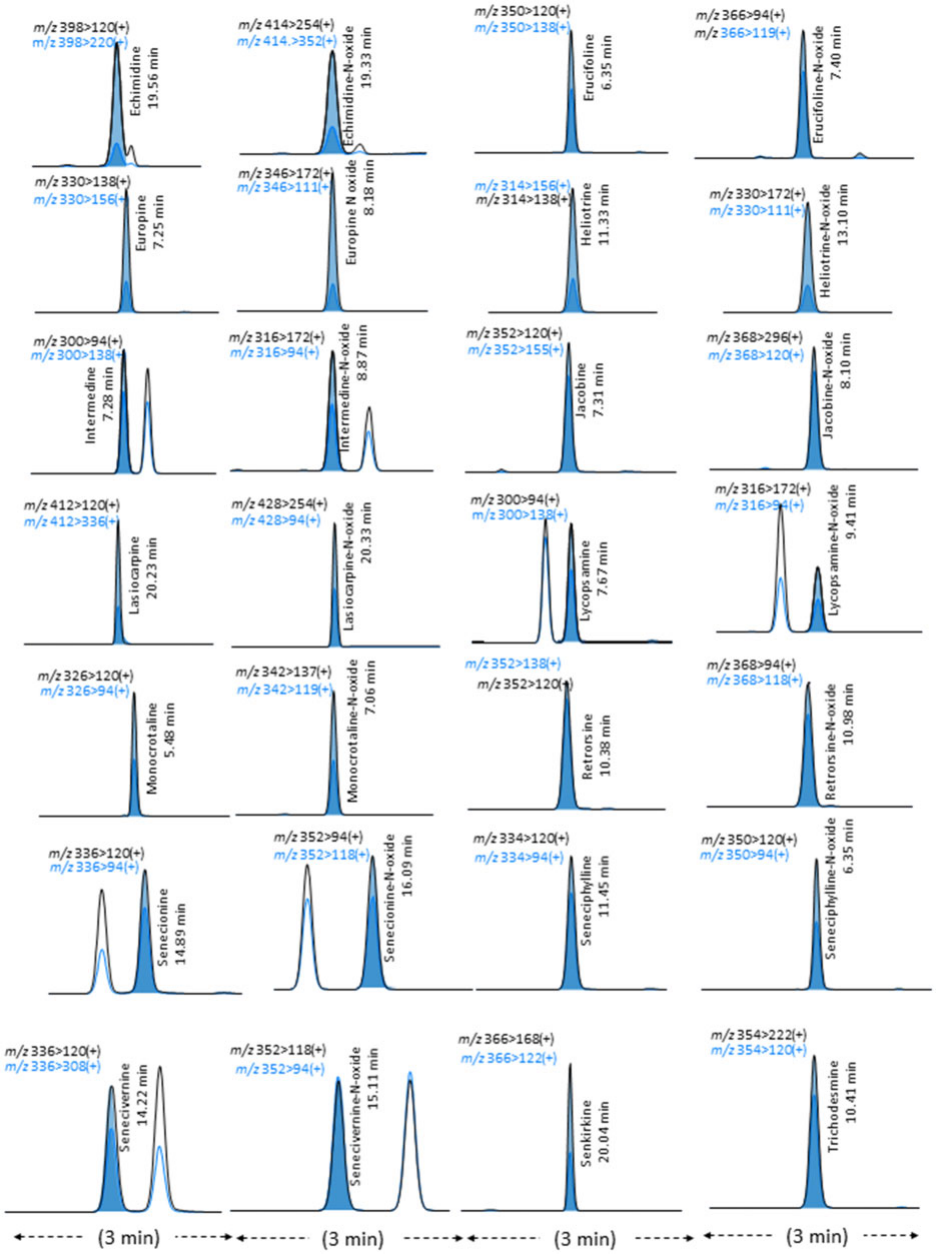
Chromatograms of the 28 PAs extracted from the proposed LC-MS/MS analysis.

The study established appropriate quantitative ions and confirmation ions in the MRM mode for the identification and quantitative analysis of PAs in herbal medicines (Table 1). The MRM conditions established in the present study were slightly different in terms of the *m/z* values in quantitative ions and confirmation ions from those of the BfR method in certain PAs. The MRM conditions had a great selectivity against the 28 PAs, as shown in Figure 3. On the basis of these results obtained through adjusting the elution solution and extraction solvent and SPE cartridge etc., this study suggests that the proposed method could obtain more improved recovery and higher sensitivity than the BfR method.

### Matrix Effect

Certain matrixes can cause significant interference in quantification analyses, affecting the values of recovery rates. Such matrix effects (MEs) can result in ion enhancement or suppression in LC-MS/MS analyses (38). As most herbal medicines are natural plants that contain various chemical compounds, they may influence the quantitative analysis of PAs. Hence, the following study examined the MEs of each PA in the five kinds of herbal medicines. The matrix effects were investigated by the following categories: *(1)* high signal suppression (−50% > ME) and moderate signal suppression (−50% < ME > −20%); *(2)* no matrix effect (−20% < ME > 20%); *(3)* moderate signal enhancement (20% < ME > 50%) and high signal enhancement (ME > 50%) (38). In other words, the ME values from −20% to 20% were assumed as reasonable values indicating minor effects. The results of the ME evaluation are summarized in Tables 2–6. In Atractylodis Rhizoma Alba, the six PAs in total (Heliotrine, Lycopasmine, Retrorsine N-oxide, Senecionine, Seneciphllyine, and Senecivernine) were observed to record their overestimation by the method performance. In contrast, most of the other PA compounds were evaluated for their suppression. In Chrysanthmi Flos, Europine indicated substantial enhancement, while the others, except for Erucifoline, Lasiocarpine, Lasiocarpine N-oxide, and Senkirkine, were estimated to induce suppression. In Leonurus Herb, the five PA compounds, such as Erucifoline, Europine, Intermedine N-oxide, Jacobine N-oxide, and Senecivernine N-oxide, demonstrated overestimation and significant enhancement, especially for the Europine. However, most of the other PAs in Leonurus Herb induced suppression. In Gastrodiae Rhizoma, the nine PAs were observed to occur high enhancement. Meanwhile, 15 PA compounds were observed to result in suppression. In Glycyrrhizae Radix et Rhizoma, the four PA compounds (Heliotrine, Herlotrine N-oxide, Retrorsine N-oxide, and Senecionine) led to remarkable improvement. At the same time, the other, except for the 3 PAs, triggered the suppression. These results revealed that the ionization of most of the 28 PA compounds in the LC-MS/MS analysis was affected by the herbal medicine matrixes with a different pattern because they have various components in plant species and families. As many PAs in five herbal medications occurred in the ME, this study conducted a matrix-matched analysis to reduce the interference of the ionization of analytes caused by the matrix components. In conclusion, as most herbal medicines used are natural raw plants possessing various components, it is considered necessary to verify whether the LC-MS/MS methods for PA quantification could be applied for the target herbal medicines even if a validated analytical method exists.

### Linearity and Sensitivity

The linear relationship between the chromatographic peak area and analyte concentration was assessed by the coefficient of determination (r^2^) obtained after the matrix-matched analysis. This study supports that the developed method disclosed the significant linearity, as the correlation coefficients (r^2^) for the 28 PA compounds were higher than 0.998 (not given) in the tested ranges listed in Tables 2–6. The LOD and LOQ were determined as the analyte concentrations that gave peak heights of at least 3–10 times higher than the noise level of the baseline, respectively. The LOD and LOQ of 28 PAs are summarized in Tables 2–6. The LOQ values of the 28 PAs were in a range between 0.1–6.5 μg/kg in Atractylodis Rhizoma Alba, 0.1–10.1 μg/kg in Chrysanthmi Flos, 0.1–5.5 μg/kg in Leonurus Herb, 0.1–9.1 μg/kg in Gastrodiae Rhizoma, and 0.1–10.5 μg/kg in Glycyrrhizae Radix et Rhizoma, respectively. Considering these results, the proposed LC-MS/MS showed sufficient sensitivity to determine the 28 PAs in the herbal medicines used in this study.

### Recovery and Precision

The recoveries and RSDs (relative standards deviations) of PAs were evaluated through LC-MS/MS analysis of the herbal medicine samples spiked the mixed standard solutions with three different concentrations (low, medium, high level). The recoveries and RSDs are depicted in Tables 2–6. The recoveries of 28 PAs obtained by the proposed LC-MS/MS analysis ranged between 72.5 and 123.7% forAtractylodis Rhizoma Alba, 70.6 and 151.7% for Chrysanthmi Flos, 80.6 and 130.9% for Leonuri Herba, 70.3 and 122.9% for Gastrodiae Rhizoma, and 71.8 and 106.9% for Glycyrrhizae Radix. The recoveries of senecionine in Chrysanthmi Flos were 120.6, 103.5, and 151.7% at low, medium, and high concentration levels and their standard deviations were 4.5, 8.6, and 10.0%, respectively.

The recoveries of the samples with low and medium concentrations were shown to be within the acceptable recovery range, even if the Chrysanthmi Flos samples with high concentrations indicated overestimated recovery values. The intra-day RSDs, reflecting the repeatability, ranged from 1.9 to 16.2% for Atractylodis Rhizoma Alba, 1.5 to 13.2% for Chrysanthmi Flos, 1.7 to 23.2% for Leonuri Herba, 1.1 to 9.8% for Gastrodiae Rhizoma, and 0.7 to 18.1% for Glycyrrhizae Radix. The inter-day RSDs, representing the reproducibility, ranged from 3.8 to 13.5% for Atractylodis Rhizoma Alba, 1.0 to 12.2% for Chrysanthmi Flos, 2.6 to 25.7% for Leonuri Herba, 1.7 to 19.0% for Gastrodiae Rhizoma, and 2.5 to 17.1% for Glycyrrhizae Radix. For most PAs, except for a few compounds in the herbal medicines used in this study, RSD values had entirely reasonable and satisfactory values in the herbal medicines. For test method validation, the AOAC guidelines require reference criteria such as a recovery rate between 70–125%, repeatability precision (intra-day RSDs) <15%, and reproducibility precision (inter-day RSDs) <32%. According to the recovery and RSD results obtained through the validation study, the current analytical method presented valid reproducibility and accuracy to determine the PAs in the five herbal medicines.

### Cross-Validation

The inter-laboratory tests were conducted on four samples to support the validity of the proposed LC-MS/MS analysis method for the determination of 28 PAs. The cross-validation assessed the selectivity, linearity, LOD, LOQ, recovery, and precisions of this method. As a result, any interfering peaks were not exhibited at the chromatographs obtained from the extraction using the proposed solvent in five herbal medicines used in this study. The linearity was >0.99 in all cases. The recoveries of Chrysanthmi Flos, Glycyrrhizae Radix et Rhizoma, Atractylodis Rhizoma Alba, Leonurus Herb, and Gastrodiae Rhizoma were found in the ranges of 72.4–120.4%, 70.4–117.0%. 70.1–119.2%, 71.5–115.5%, and 71.1–118.4%, respectively. The RSD values of most samples were found to be within acceptable ranges (Supplemental Data 2–6). As the recoveries and RSDs of most samples gained from cross-validation satisfied the acceptance criteria of AOAC, the proposed LC-MS/MS analysis method was assumed to be available for determining the 28 PAs in five herbal medicines.

### System Suitability

The system suitability was ensured by checking whether the RSD values can be collected after five replicates measuring the standard solution. The results were within the acceptance criteria (40), i.e., RSDs of the area were less than 5% (Supplemental Data 1). Also, this study confirmed the system suitability of the LC-MS/MS by examining these outcomes in retention time, tailing factor, number of theoretical plates, resolution, peak width, and height equivalent to a theoretical plate (the data were not shown).

### Monitoring of PAs in Herbal Medicines

The developed simultaneous LC-MS/MS method was applied to determine the contents of 28 PAs in 50 herbal medicine samples purchased from Korean herbal medicine markets. Ten samples of each kind of herbal medicine, such as Glycyrrhizae Radix et Rhizoma, Atractylodis Rhizoma Alba, Leonurus Herb, Gastrodiae Rhizoma, and Chrysanthmi Flos, were used for the monitoring study. Most herbal medicines did not contain any of the 28 PAs or had a content of less than the LOQ. Only Leonuri Herbal samples contained two PAs of Retrorsine and Senkirkine. Leonuri Herba was found to have Retrorsine of the concentration range of 84.7–120.9 μg/kg, whereas all Leonuri Herba contained Senkirkine in the concentration range of 60.9–170.7 μg/kg.

Edgar et al. (12) reported that Moncrotaline in Crotalaria species was identified by GC-electron impact MS. Mulder (39) further claimed that PAs could be detected with a mean concentration of 991.0 μg/kg in some herbal drugs. Moreover, Letsyo et al. (40) investigated the presence of PAs in 98 patronized herbal medicines from six popular German retail supermarkets/drugstores and pharmacies using LC-ESI-MS/MS. They reported that about 63% of the herbal medicines consisted of PAs, and the average PA concentration of the samples was 201 μg/kg. A herbal medicinal product with Hypericum perforatum L. (St. John’s Wort) as an active ingredient demonstrated the highest PA concentration (3270 μg/kg).

According to the monitoring results obtained from another laboratory in MFDS, Leonurus Herba contained Trichodesmine (1/8) of 20 μg/kg and Heliotrine N-oxide (2/8) of 1.0 μg/kg in eight samples. Various herbal medicinal products manufactured using herbal preparations or herbal medicines are distributed and sold at herbal medicine stores or drugstores. Considering the time and expense of each trial, it did not appear to be efficient to monitor the presence of the PAstargeting all herbal medicinal products. In these respects, the potential herbal medicines that may contain the PAs using the verified LC-MS/MS method should essentially be searched. Based on these results, the residual amount of PAs in the herbal medicinal products using the positive herbal medicines should also be tracked to reduce the health risk PAs can potentially cause. The residual PA amount against herbal medicinal products using Leonurus Herba can be considered to be monitored in further studies.

## Conclusions

The current study primarily aimed to develop and validate a precise and accessible LC-MS/MS method to determine 28 PAs in five types of herbal medicines. In addition, this study examined the contamination status of PAs using this method. The overall outcomes improved the LC-MS/MS method developed by MFDS, Korea, to determine PAs in tea by changing the composition of the elution and the mobile phase solution. The proposed LC-MS/MS procedure was coupled with an MCX-SPE cleanup to trace the PAs in five kinds of herbal medicines (Atractylodis Rhizoma Alba, Leonuri Herba, Gastrodiae Rhizoma, and Glycyrrhizae Radix). According to the values of recovery and sensitivity obtained from the PA analysis, the MCX-SPE cleanup approach effectively removed interfering materials in these herbal medicines. The proposed analysis method for determining the 28 PAs showed entirely acceptable accuracy and precision in herbal medicines. In the monitoring study, only Leonuri Herba contained two PAs, which were Retrorsine (4/10) of 80–120 μg/kg and Senkirkine (10/10) of 60–170 μg/kg. These findings suggest that the developed LC-MS/MS analysis method can be successfully applied to trace amounts of PAs in herbal medicines such as Atractylodis Rhizoma Alba, Leonuri Herba, Gastrodiae Rhizoma, and Glycyrrhizae Radix.

## Supplemental Information

Supplemental information is available on the *J. AOAC Int*. website.

## CrediT Author Statement 

Kyung Yuk Ko: Writing-original draft, investigation, methodology, conceptualization. Se, Hee Jeong: Formal analysis, visualization, data curation. Eun Young Choi: Methodology, validation. Jonghwan Kim: Conceptualization, methodology. Chulhyun Lee: Investigation, software. Juhye Kang, project administration. Sooyeul Cho: Supervision, project administration.

## Funding

This research was supported by a grant (19171MFDS193) from the Ministry of Food and Drug Safety in 2019. The authors would like to thank Jihyun Lee and Youngmin Hong of the Shimadzu Scientific Korea for assistance with the LC-MS/MS analysis.

## Supplementary Material

qsab098_Supplementary_Data
