# Supplementary material for: LC-ESI-MS/MS Simultaneous Analysis Method Coupled with Cation-Exchange Solid-Phase Extraction for Determination of Pyrrolizidine Alkaloids on Five Kinds of Herbal Medicines
Source: J AOAC Int. 2021 Jul 23;104(6):1514–25. doi: 10.1093/jaoacint/qsab098 (PMC8665765; doi:10.1093/jaoacint/qsab098)
Supplement: qsab098_Supplementary_Data [file qsab098_supplementary_data.docx]

Supplement 1. RSD values of peak area in each PA obtained by five replicates with mixed standard solution

| No. | Compound | Peak area | | | | | RSD (%) |
| --- | --- | --- | --- | --- | --- | --- | --- |
|  |  | 1 | 2 | 3 | 4 | 5 |  |
| 1 | Echimidine | 961209 | 972058 | 968809 | 975624 | 952945 | 0.9 |
| 2 | Echimidine-N-oxide | 707303 | 750838 | 734105 | 718050 | 708383 | 2.6 |
| 3 | Erucifoline | 222904 | 230311 | 209988 | 212299 | 220050 | 3.8 |
| 4 | Erucifoline-N-oxide | 195348 | 208405 | 196535 | 202134 | 203978 | 2.7 |
| 5 | Europine | 168663 | 160761 | 162236 | 175613 | 167215 | 3.5 |
| 6 | Europine-N-oxide | 840840 | 920635 | 884243 | 845569 | 832477 | 4.3 |
| 7 | Heliotrine | 1332974 | 1352565 | 1330963 | 1332621 | 1333026 | 0.7 |
| 8 | Heliotrine-N-oxide | 1772595 | 1814824 | 1811994 | 1780680 | 1767439 | 1.2 |
| 9 | Intermedine | 495241 | 483283 | 465607 | 465015 | 460851 | 3.1 |
| 10 | Intermedine-N-oxide | 902315 | 829158 | 830549 | 858805 | 869477 | 3.5 |
| 11 | Jacobine | 110726 | 120600 | 115976 | 119699 | 114424 | 3.5 |
| 12 | Jacobine-N-oxide | 449263 | 466304 | 454194 | 479786 | 434286 | 3.8 |
| 13 | Lasiocarpine | 668966 | 711930 | 719816 | 661923 | 675669 | 3.8 |
| 14 | Lasiocarpine-N-oxide | 53023 | 53809 | 57731 | 53723 | 52890 | 3.7 |
| 15 | Lycopsamine | 356276 | 371105 | 358124 | 359840 | 350759 | 2.1 |
| 16 | Lycopsamine-N-oxide | 80475 | 86316 | 86316 | 88982 | 86405 | 3.7 |
| 17 | Monocrotaline | 326729 | 330606 | 327501 | 325871 | 321141 | 1.0 |
| 18 | Monocrotaline-N-oxide | 203796 | 214407 | 206415 | 197187 | 197366 | 3.5 |
| 19 | Retrorsine | 136102 | 133746 | 136005 | 129014 | 124627 | 3.8 |
| 20 | Retrorsine-N-oxide | 126015 | 126920 | 126325 | 131096 | 132920 | 2.4 |
| 21 | Senecionine | 226518 | 216917 | 222176 | 219256 | 222780 | 1.6 |
| 22 | Senecionine-N-oxide | 177709 | 184611 | 183799 | 179687 | 180837 | 1.6 |
| 23 | Seneciphylline | 237613 | 244320 | 239262 | 235137 | 240007 | 1.4 |
| 24 | Seneciphylline-N-oxide | 182985 | 180140 | 179621 | 193396 | 190503 | 3.4 |
| 25 | Senecivernine | 276412 | 267479 | 277531 | 277792 | 286538 | 2.4 |
| 26 | Senecivernine-N-oxide | 219201 | 205854 | 211009 | 207089 | 214572 | 2.6 |
| 27 | Senkirkine | 492073 | 474230 | 505039 | 490888 | 475346 | 2.6 |
| 28 | Trichodesmine | 528232 | 506142 | 519095 | 505362 | 501541 | 2.2 |

Supplement data 2. The results of recovery and RSD of Chrysanthmi Flos obtained from cross-validation (n=7)

| No. | PA Compound | LC^*^ | | MC^**^ | | HC^***^ | |
| --- | --- | --- | --- | --- | --- | --- | --- |
|  |  | Recovery(%) | RSD | Recovery(%) | RSD | Recovery(%) | RSD |
| 1 | Echimidine | 91.1±14.8 | 16.2 | 97.3±9.5 | 9.7 | 103.0±4.4 | 4.2 |
| 2 | Echimidine-N-oxide | 109.9±26.5 | 24.1 | 111.8±14.2 | 12.7 | 120.4±5.1 | 4.2 |
| 3 | Erucifoline | 87.2±16.2 | 18.5 | 82.3±13.5 | 16.4 | 72.6±4.2 | 5.8 |
| 4 | Erucifoline-N-oxide | 95.5±19.5 | 20.4 | 92.1±12.8 | 13.9 | 83.6±5.3 | 6.4 |
| 5 | Europine | 85.8±15.3 | 17.8 | 80.1±11.9 | 14.9 | 72.4±6.3 | 8.7 |
| 6 | Europine-N-oxide | 95.6±11.8 | 12.3 | 102.2±13.8 | 13.5 | 98.6±4.4 | 4.4 |
| 7 | Heliotrine | 91.7±16.8 | 18.3 | 85.2±8.1 | 9.5 | 82.7±6.7 | 8.1 |
| 8 | Heliotrine-N-oxide | 95.0±19.1 | 20.1 | 87.3±11.2 | 12.8 | 87.3±6.1 | 7.0 |
| 9 | Intermedine | 82.6±11.4 | 13.8 | 85.7±12.2 | 14.3 | 76.2±6.1 | 8.0 |
| 10 | Intermedine-N-oxide | 103.2±14.9 | 14.4 | 95.9±13.8 | 14.4 | 89.3±4.7 | 5.3 |
| 11 | Jacobine | 83.2±8.5 | 10.2 | 82.9±5.0 | 6.0 | 73.3±2.4 | 3.3 |
| 12 | Jacobine-N-oxide | 116.8±15.4 | 13.1 | 104.9±17.1 | 16.4 | 95.8±5.7 | 5.9 |
| 13 | Lasiocarpine | 107.9±10.0 | 9.2 | 96.4±11.4 | 11.9 | 98.6±12.1 | 12.3 |
| 14 | Lasiocarpine-N-oxide | 106.5±14.2 | 13.4 | 115.0±4.8 | 4.2 | 117.5±5.0 | 4.3 |
| 15 | Lycopsamine | 88.3±15.2 | 17.2 | 86.3±9.6 | 11.1 | 74.7±4.9 | 6.6 |
| 16 | Lycopsamine-N-oxide | 92.5±17.2 | 18.6 | 86.6±6.3 | 7.3 | 85.5±5.4 | 6.3 |
| 17 | Monocrotaline | 107.6±16.5 | 15.4 | 99.5±16.5 | 16.6 | 79.2±9.0 | 11.4 |
| 18 | Monocrotaline-N-oxide | 94.3±13.8 | 14.6 | 94.0±10.1 | 10.7 | 81.3±5.8 | 7.2 |
| 19 | Retrorsine | 82.6±15.5 | 18.8 | 76.3±12.1 | 15.8 | 72.7±4.3 | 5.9 |
| 20 | Retrorsine-N-oxide | 100.8±18.5 | 18.3 | 91.8±11.9 | 13.0 | 90.3±5.9 | 6.6 |
| 21 | Senecionine | 93.2±16.7 | 17.9 | 76.5±8.7 | 11.4 | 82.3±5.5 | 6.7 |
| 22 | Senecionine-N-oxide | 105.4±18.2 | 17.2 | 99.8±9.4 | 9.4 | 97.1±7.4 | 7.6 |
| 23 | Seneciphylline | 81.0±4.2 | 5.2 | 73.3±2.5 | 3.4 | 74.7±4.7 | 6.2 |
| 24 | Seneciphylline-N-oxide | 81.9±15.5 | 18.9 | 77.5±7.9 | 10.1 | 75.7±4.8 | 6.3 |
| 25 | Senecivernine | 98.7±15.5 | 15.7 | 90.8±6.1 | 6.8 | 77.2±4.6 | 6.0 |
| 26 | Senecivernine-N-oxide | 106.6±21.9 | 20.6 | 100.4±10.9 | 10.9 | 103.8±8.9 | 8.5 |
| 27 | Senkirkine | 120.2±23.0 | 19.1 | 112.7±6.5 | 5.8 | 98.9±6.6 | 6.7 |
| 28 | Trichodesmine | 90.9±16.8 | 18.5 | 87.6±10.6 | 12.1 | 89.0±6.6 | 7.4 |

^*^LC: low concentration, ^**^MC: medium concentraion, ^***^ HC: high concentraion

Supplement data 3. The results of recovery and RSD of Glycyrrhizae Radix et Rhizoma obtained from cross-validation (n=7)

| No. | PA compound | LC^*^ | | MC^**^ | | HC^***^ | |
| --- | --- | --- | --- | --- | --- | --- | --- |
|  |  | Recovery(%) | RSD | Recovery(%) | RSD | Recovery(%) | RSD |
| 1 | Echimidine | 79.8 ± 1.2 | 1.6 | 80.7±2.7 | 3.3 | 88.8±1.7 | 2.0 |
| 2 | Echimidine-N-oxide | 107.3 ± 2.1 | 1.9 | 107.3±4.6 | 4.3 | 117.0±3.4 | 2.9 |
| 3 | Erucifoline | 93.2 ± 2.6 | 2.8 | 93.4±4.0 | 4.2 | 102.2±3.8 | 3.7 |
| 4 | Erucifoline-N-oxide | 116.9 ± 8.1 | 7.0 | 115.1±7.4 | 6.5 | 119.8±4.9 | 4.1 |
| 5 | Europine | 76.5 ± 2.6 | 3.3 | 82.8±6.8 | 8.2 | 99.7±6.1 | 6.1 |
| 6 | Europine-N-oxide | 90.5 ± 1.8 | 2.0 | 93.8±3.9 | 4.1 | 103.1±5.5 | 5.3 |
| 7 | Heliotrine | 89.9 ± 1.1 | 1.3 | 89.8±4.6 | 5.1 | 98.9±2.2 | 2.3 |
| 8 | Heliotrine-N-oxide | 101.6 ± 1.6 | 1.6 | 101.3±4.6 | 4.5 | 105.7±2.9 | 2.8 |
| 9 | Intermedine | 95.3 ± 3.9 | 4.1 | 97.6±5.8 | 6.0 | 104.8±2.3 | 2.2 |
| 10 | Intermedine-N-oxide | 94.5 ± 1.8 | 1.9 | 92.6±4.3 | 4.6 | 99.5±3.5 | 3.5 |
| 11 | Jacobine | 82.3 ± 2.1 | 2.5 | 90.3±2.7 | 3.0 | 99.0±2.9 | 3.0 |
| 12 | Jacobine-N-oxide | 111.8 ± 4.0 | 3.6 | 111.8±5.1 | 4.5 | 119.5±4.2 | 3.5 |
| 13 | Lasiocarpine | 70.9 ± 0.9 | 1.3 | 74.6±3.5 | 4.7 | 70.4±1.5 | 2.2 |
| 14 | Lasiocarpine-N-oxide | 91.6 ± 1.9 | 2.1 | 92.6±3.5 | 3.8 | 87.8±3.0 | 3.4 |
| 15 | Lycopsamine | 94.5 ± 3.9 | 4.2 | 96.6±3.8 | 3.9 | 102.2±3.1 | 3.1 |
| 16 | Lycopsamine-N-oxide | 93.8 ± 6.8 | 7.3 | 96.1±3.6 | 3.8 | 100.4±3.7 | 3.7 |
| 17 | Monocrotaline | 91.1 ± 1.5 | 1.7 | 88.5±3.2 | 3.7 | 92.7±4.1 | 4.4 |
| 18 | Monocrotaline-N-oxide | 100.3 ± 2.9 | 2.9 | 102.9±2.4 | 2.3 | 110.9±4.1 | 3.7 |
| 19 | Retrorsine | 80.7 ± 3.6 | 4.4 | 82.8±2.6 | 3.1 | 91.8±3.0 | 3.3 |
| 20 | Retrorsine-N-oxide | 105.8 ± 1.3 | 1.3 | 104.8±4.4 | 4.2 | 115.2±3.3 | 2.9 |
| 21 | Senecionine | 71.3 ± 3.0 | 4.2 | 74.2±1.7 | 2.3 | 81.2±2.4 | 3.0 |
| 22 | Senecionine-N-oxide | 102.9 ± 1.1 | 1.0 | 102.4±3.9 | 3.8 | 106.7±3.0 | 2.8 |
| 23 | Seneciphylline | 74.8 ± 1.9 | 2.6 | 76.5±2.1 | 2.7 | 84.7±2.5 | 2.9 |
| 24 | Seneciphylline-N-oxide | 92.2 ± 2.7 | 2.9 | 91.7±2.6 | 2.8 | 95.9±3.9 | 4.1 |
| 25 | Senecivernine | 72.6 ± 2.5 | 3.4 | 72.2±2.1 | 2.9 | 78.5±2.4 | 3.0 |
| 26 | Senecivernine-N-oxide | 108.6 ± 1.5 | 1.4 | 108.0±3.1 | 2.8 | 110.7±3.6 | 3.3 |
| 27 | Senkirkine | 102.8 ± 2.1 | 2.0 | 93.0±2.6 | 2.8 | 95.0±1.3 | 1.3 |
| 28 | Trichodesmine | 87.9 ± 2.3 | 2.6 | 87.3±2.4 | 2.7 | 96.3±3.4 | 3.6 |

^*^LC: low concentration, ^**^MC: medium concentraion, ^***^ HC: high concentraion

Supplement data 4. The results of recovery and RSD of Atractylodis Rhizoma Alba obtained from cross-validation (n=7)

| No. | PA Compound | LC^*^ | | MC^**^ | | HC^***^ | |
| --- | --- | --- | --- | --- | --- | --- | --- |
|  |  | Recovery(%) | RSD | Recovery(%) | RSD | Recovery(%) | RSD |
| 1 | Echimidine | 86.6±3.4 | 3.9 | 91.3±1.3 | 1.5 | 103.2±3.7 | 3.6 |
| 2 | Echimidine-N-oxide | 83.5±3.5 | 4.2 | 87.4±1.4 | 1.6 | 97.8±2.8 | 2.9 |
| 3 | Erucifoline | 86.0±4.0 | 4.7 | 89.4±1.3 | 1.5 | 101.6±4.1 | 4.0 |
| 4 | Erucifoline-N-oxide | 81.3±4.2 | 5.1 | 88.8±1.3 | 1.5 | 101.0±1.5 | 1.5 |
| 5 | Europine | 72.6±1.2 | 1.7 | 70.4±3.9 | 5.5 | 70.1±0.9 | 1.3 |
| 6 | Europine-N-oxide | 86.0±3.8 | 4.4 | 90.1±6.1 | 6.8 | 90.8±2.7 | 3.0 |
| 7 | Heliotrine | 77.9±2.9 | 3.7 | 83.2±3.3 | 3.9 | 85.1±2.1 | 2.5 |
| 8 | Heliotrine-N-oxide | 88.6±3.0 | 3.4 | 93.3±2.0 | 2.2 | 97.6±2.9 | 3.0 |
| 9 | Intermedine | 76.6±3.9 | 5.0 | 78.7±2.8 | 3.5 | 86.2±2.8 | 3.3 |
| 10 | Intermedine-N-oxide | 85.1±3.0 | 3.5 | 88.5±2.4 | 2.7 | 93.8±3.1 | 3.3 |
| 11 | Jacobine | 83.9±4.8 | 5.7 | 90.1±3.5 | 3.9 | 103.2±3.7 | 3.6 |
| 12 | Jacobine-N-oxide | 88.5±3.3 | 3.7 | 94.3±0.8 | 0.8 | 99.2±3.3 | 3.4 |
| 13 | Lasiocarpine | 93.9±4.0 | 4.3 | 89.2±2.6 | 2.9 | 112.1±5.0 | 4.4 |
| 14 | Lasiocarpine-N-oxide | 94.0±3.5 | 3.7 | 96.1±2.7 | 2.8 | 119.2±5.8 | 4.8 |
| 15 | Lycopsamine | 81.4±4.2 | 5.2 | 79.2±2.8 | 3.6 | 89.6±3.4 | 3.7 |
| 16 | Lycopsamine-N-oxide | 83.9±3.9 | 4.6 | 89.4±2.9 | 3.2 | 95.3±4.1 | 4.3 |
| 17 | Monocrotaline | 80.4±3.2 | 3.9 | 79.5±1.2 | 1.5 | 92.5±3.5 | 3.7 |
| 18 | Monocrotaline-N-oxide | 77.7±4.2 | 5.4 | 79.8±2.3 | 2.8 | 92.9±4.9 | 5.2 |
| 19 | Retrorsine | 83.8±5.5 | 6.5 | 86.4±3.8 | 4.4 | 104.2±3.6 | 3.4 |
| 20 | Retrorsine-N-oxide | 91.1±3.3 | 3.7 | 96.1±2.2 | 2.3 | 107.8±4.0 | 3.7 |
| 21 | Senecionine | 88.2±3.5 | 4.0 | 87.9±2.0 | 2.2 | 110.8±3.6 | 3.2 |
| 22 | Senecionine-N-oxide | 90.7±3.3 | 3.6 | 99.6±1.1 | 1.1 | 119.2±4.2 | 3.5 |
| 23 | Seneciphylline | 80.5±2.8 | 3.5 | 81.1±0.4 | 0.5 | 100.2±4.7 | 4.7 |
| 24 | Seneciphylline-N-oxide | 87.6±5.1 | 5.9 | 87.8±2.5 | 2.9 | 101.0±4.3 | 4.3 |
| 25 | Senecivernine | 81.3±3.5 | 4.3 | 84.4±0.9 | 1.1 | 107.9±3.8 | 3.6 |
| 26 | Senecivernine-N-oxide | 89.2±4.4 | 5.0 | 96.6±2.7 | 2.8 | 119.0±3.0 | 2.6 |
| 27 | Senkirkine | 99.7±6.7 | 6.7 | 96.7±1.5 | 1.6 | 117.7±4.0 | 3.4 |
| 28 | Trichodesmine | 83.8±4.5 | 5.3 | 86.8±1.1 | 1.3 | 107.6±4.9 | 4.6 |

^*^LC: low concentration, ^**^MC: medium concentraion, ^***^ HC: high concentraion

Supplement data 5. The results of recovery and RSD of Leonurus Herb obtained from cross-validation (n=7)

| No. | PA compound | LC^*^ | | MC^**^ | | HC^***^ | |
| --- | --- | --- | --- | --- | --- | --- | --- |
|  |  | Recovery(%) | RSD | Recovery(%) | RSD | Recovery(%) | RSD |
| 1 | Echimidine | 85.9±7.8 | 9.1 | 88.6±9.5 | 10.7 | 103.3±7.3 | 7.1 |
| 2 | Echimidine-N-oxide | 100.4±9.2 | 9.1 | 104.4±12.4 | 11.9 | 115.2±6.0 | 5.2 |
| 3 | Erucifoline | 74.7±6.2 | 8.3 | 73.0±13.5 | 18.5 | 72.1±3.8 | 5.3 |
| 4 | Erucifoline-N-oxide | 89.1±13.3 | 15.0 | 97.7±11.5 | 11.7 | 95.9±8.6 | 9.0 |
| 5 | Europine | 77.1±8.8 | 11.4 | 82.0±16.2 | 19.8 | 78.6±7.3 | 9.3 |
| 6 | Europine-N-oxide | 85.3±8.1 | 9.5 | 92.4±15.5 | 16.8 | 91.6±8.8 | 9.6 |
| 7 | Heliotrine | 90.6±5.4 | 6.0 | 92.1±12.9 | 14.1 | 87.0±9.3 | 10.7 |
| 8 | Heliotrine-N-oxide | 90.1±8.6 | 9.5 | 101.5±14.2 | 14 | 106.4±9.0 | 8.5 |
| 9 | Intermedine | 80.1±4.9 | 6.1 | 91.5±18.5 | 20.2 | 93.7±12.1 | 12.9 |
| 10 | Intermedine-N-oxide | 62.3±4.4 | 7.1 | 81.2±17.9 | 22 | 82.6±9.2 | 11.2 |
| 11 | Jacobine | 81.8±10.6 | 13 | 84.7±12.3 | 14.6 | 87.9±7.8 | 8.9 |
| 12 | Jacobine-N-oxide | 101.6±13.5 | 13.3 | 101.7±9.5 | 9.4 | 99.2±10.6 | 10.6 |
| 13 | Lasiocarpine | 86.7±13.2 | 15.2 | 89.3±9.8 | 11 | 98.4±9.9 | 10.1 |
| 14 | Lasiocarpine-N-oxide | 99.5±4.4 | 4.4 | 111.6±7.3 | 6.6 | 116.5±7.3 | 6.3 |
| 15 | Lycopsamine | 78.3±9.4 | 12 | 86.0±11.3 | 13.1 | 87.3±6.6 | 7.5 |
| 16 | Lycopsamine-N-oxide | 99.3±7.8 | 7.8 | 109.5±18.7 | 17.1 | 101.9±9.0 | 8.9 |
| 17 | Monocrotaline | 92.2±8.1 | 8.7 | 92.7±20.7 | 22.4 | 88.9±9.0 | 10.1 |
| 18 | Monocrotaline-N-oxide | 82.4±6.1 | 7.4 | 92.9±14.8 | 15.9 | 91.1±10.0 | 11.0 |
| 19 | Retrorsine | 79.5±13.5 | 17 | 82.1±12.9 | 15.8 | 82.5±6.2 | 7.5 |
| 20 | Retrorsine-N-oxide | 98.3±10.3 | 10.4 | 102.9±11.8 | 11.5 | 108.7±10.3 | 9.5 |
| 21 | Senecionine | 78.4±8.8 | 11.2 | 73.5±9.5 | 12.9 | 83.7±5.9 | 7.1 |
| 22 | Senecionine-N-oxide | 97.5±11.8 | 12.1 | 102.5±7.5 | 7.3 | 108.7±8.9 | 8.1 |
| 23 | Seneciphylline | 78.8±6.1 | 7.7 | 71.5±9.3 | 13.1 | 75.7±6.5 | 8.6 |
| 24 | Seneciphylline-N-oxide | 73.7±9.1 | 12.3 | 73.8±13.4 | 18.1 | 73.7±6.5 | 8.8 |
| 25 | Senecivernine | 77.8±9.4 | 12.1 | 72.9±6.2 | 8.5 | 76.3±9.6 | 12.6 |
| 26 | Senecivernine-N-oxide | 97.8±7.0 | 7.2 | 105.2±8.4 | 8.0 | 109.7±8.7 | 7.9 |
| 27 | Senkirkine | 115.5±14.5 | 12.6 | 109.6±6.7 | 6.1 | 103.6±4.4 | 4.2 |
| 28 | Trichodesmine | 76.1±2.4 | 3.2 | 88.3±11.7 | 13.3 | 98.4±8.8 | 9.0 |

^*^LC: low concentration, ^**^MC: medium concentraion, ^***^ HC: high concentraion

Supplement data 6. The results of recovery and RSD of Gastrodiae Rhizoma obtained from cross-validation (n=7)

| No. | PA compound | LC^*^ | | MC^**^ | | HC^***^ | |
| --- | --- | --- | --- | --- | --- | --- | --- |
|  |  | Recovery(%) | RSD | Recovery(%) | RSD | Recovery(%) | RSD |
| 1 | Echimidine | 102.8±5.0 | 4.9 | 99.8±5.3 | 5.3 | 116.6±1.7 | 1.4 |
| 2 | Echimidine-N-oxide | 72.3±1.9 | 2.6 | 73.7±6.2 | 8.4 | 79.2±6.5 | 8.2 |
| 3 | Erucifoline | 94.2±4.3 | 4.6 | 93.6±7.1 | 7.6 | 104.0±4.2 | 4.0 |
| 4 | Erucifoline-N-oxide | 71.7±2.0 | 2.8 | 70.1±2.9 | 4.1 | 78.8±4.1 | 5.2 |
| 5 | Europine | 86.8±2.4 | 2.7 | 87.3±4.1 | 4.7 | 101.4±4.1 | 4.0 |
| 6 | Europine-N-oxide | 84.6±2.4 | 2.8 | 87.4±5.3 | 6.1 | 90.1±3.9 | 4.3 |
| 7 | Heliotrine | 114.1±9.0 | 7.9 | 113.0±8.8 | 7.8 | 116.1±2.2 | 1.9 |
| 8 | Heliotrine-N-oxide | 86.0±1.5 | 1.8 | 84.5±6.7 | 7.9 | 96.6±4.6 | 4.8 |
| 9 | Intermedine | 87.8±5.8 | 6.6 | 89.6±1.8 | 2.0 | 103.6±1.8 | 1.7 |
| 10 | Intermedine-N-oxide | 82.9±1.8 | 2.1 | 80.4±3.1 | 3.9 | 85.8±3.1 | 3.6 |
| 11 | Jacobine | 85.4±3.3 | 3.9 | 90.0±4.7 | 5.2 | 109.1±3.2 | 2.9 |
| 12 | Jacobine-N-oxide | 71.3±2.3 | 3.3 | 70.1±4.4 | 6.2 | 71.1±4.1 | 5.7 |
| 13 | Lasiocarpine | 92.7±2.0 | 2.1 | 87.2±3.6 | 4.1 | 94.7±3.2 | 3.4 |
| 14 | Lasiocarpine-N-oxide | 89.9±1.3 | 1.4 | 87.2±3.1 | 3.5 | 96.0±3.8 | 3.9 |
| 15 | Lycopsamine | 98.7±4.6 | 4.7 | 96.0±2.8 | 3.0 | 109.6±2.8 | 2.6 |
| 16 | Lycopsamine-N-oxide | 77.0±2.7 | 3.5 | 82.3±6.4 | 7.7 | 96.5±5.0 | 5.2 |
| 17 | Monocrotaline | 115.5±8.8 | 7.6 | 118.8±6.2 | 5.2 | 118.4±6.9 | 5.8 |
| 18 | Monocrotaline-N-oxide | 72.7±1.8 | 2.5 | 74.1±3.2 | 4.3 | 79.6±2.2 | 2.8 |
| 19 | Retrorsine | 90.8±3.8 | 4.2 | 90.6±4.7 | 5.2 | 104.7±3.9 | 3.7 |
| 20 | Retrorsine-N-oxide | 74.3±1.4 | 1.9 | 73.3±4.8 | 6.5 | 82.4±3.8 | 4.6 |
| 21 | Senecionine | 93.5±8.0 | 8.6 | 94.2±1.6 | 1.7 | 108.9±2.3 | 2.1 |
| 22 | Senecionine-N-oxide | 72.8±0.6 | 0.8 | 73.7±6.0 | 8.1 | 81.9±5.9 | 7.2 |
| 23 | Seneciphylline | 79.3±4.2 | 5.3 | 80.6±5.0 | 6.2 | 92.9±1.8 | 2.0 |
| 24 | Seneciphylline-N-oxide | 92.6±5.2 | 5.6 | 93.3±6.0 | 6.5 | 105.7±2.8 | 2.7 |
| 25 | Senecivernine | 97.0±4.0 | 4.1 | 81.4±9.7 | 11.9 | 97.2±2.5 | 2.6 |
| 26 | Senecivernine-N-oxide | 74.6±1.1 | 1.5 | 74.1±3.1 | 4.2 | 78.3±4.2 | 5.3 |
| 27 | Senkirkine | 93.2±4.9 | 5.2 | 100.6±6.6 | 6.6 | 107.7±5.2 | 4.9 |
| 28 | Trichodesmine | 81.8±2.8 | 3.4 | 84.6±2.1 | 2.5 | 94.4±4.3 | 4.5 |

^*^LC: low concentration, ^**^MC: medium concentraion, ^***^ HC: high concentraion
